# Advanced trap lateral flow immunoassay sensor for the detection of cortisol in human bodily fluids

**DOI:** 10.1038/s41598-021-02084-7

**Published:** 2021-11-19

**Authors:** Hyun-Kyung Oh, Kihyeun Kim, Jinhee Park, Hyungjun Jang, Min-Gon Kim

**Affiliations:** 1 Department of Chemistry, Gwangju Institute of Science and Technology (GIST), 123 Cheomdangawgi-ro, Buk-gu, Gwangju, 61005 Republic of Korea; 2 GMD Biotech Co. Ltd., 416, Industry-Academic Cooperation Building, 123 Cheomdangawgi-ro, Buk-gu, Gwangju, 61005 Republic of Korea

**Keywords:** Analytical biochemistry, Lab-on-a-chip, Sensors and probes, Biochemistry, Biomarkers

## Abstract

Paper-based biosensors based on lateral flow immunoassay (LFI) are promising candidates for POC diagnosis because of their ease of use and rapid target detection. However, the low sensitivity of LFI limits its application, and signal amplification has been used in numerous studies to increase its sensitivity. We developed an advanced trap LFI (*α*-trapLFI), a simple-to-use sensor, with an additional step for signal amplification. Here, signal amplification is automatically implemented following delayed release of enhancement solution induced by water-soluble polyvinyl alcohol tape. As the polyvinyl alcohol tape is exposed to water, its polymer structure is perturbed (within 5 min), allowing ions to pass through. This new sensor was designed to have a short time delay between the flow of solutions used for the immunoassay and signal amplification. The *α*-trapLFI was subsequently used to detect cortisol with high sensitivity (9.1 pg∙mL^−1^) over a broad detection range (0.01–1000 ng∙mL^−1^) in bodily fluids. Furthermore, an excellent correlation was obtained by analyzing 20 human real saliva samples using this sensor and a conventional ELISA (*R*^2^ = 0.90). The new sensor will be helpful in detecting various small molecules for simple, rapid, and portable POC diagnosis of stress disorders.

## Introduction

The stress hormone cortisol has emerged as a biomarker for the rapid diagnosis and treatment of post-traumatic stress disorders^1,2^. Excessive and persistent cortisol secretion is known to cause serious side effects, such as increased blood pressure, decreased immune response, chronic fatigue, and insomnia^3^. A recent study showed that high cortisol concentrations in patients with coronavirus disease-2019 are associated with increased mortality and a decreased average survival rate^4^. Moreover, 24-h monitoring of cortisol secretion could be useful for diagnosis and treatment psychotic and nonpsychotic major depressive disorders associated with distinct patterns of HPA axis dysregulation^5^. Thus, quantification of cortisol levels is essential for evaluating and managing mental, physical, and physiological stresses. Among the various methods of monitoring cortisol secretion, point-of-care (POC) testing is desirable because cortisol levels change depending on the surrounding environment and behavior of individuals during the day.

Biosensors based on a lateral flow immunoassay (LFI) are promising candidates for POC diagnosis, as they are easy to use by non-specialized individuals and enable rapid target detection without specialized equipment^6–8^. However, most LFI-based biosensors show low sensitivity and cannot provide quantitative results^9^. To overcome these limitations, several signal amplification methods have been evaluated for use with LFI, including the application of enzymes for additional color-developing reactions^10–13^ and application of nanoparticles for fluorescent signal enhancement^14–17^. Although these methods can improve the detection limit of the assay, several limitations prevent operational POC diagnosis, including stability issues and the requirement for extra equipment to detect fluorescent signals.

In 2011, Wei et al. suggested using metal ions to enhance the sensitivity of LFI^18^. After performing a general immunoassay using metal nanoparticles, Wei et al. amplified the signal by inducing metal nanoparticle growth through addition of a metal ion and reducing agent. Specifically, detection antibody-gold nanoparticle (AuNP) conjugates bound to the analyte were immobilized on a detection antibody-loaded membrane and then reacted with gold ions and a reducing agent in the reaction buffer to enlarge the metal nanoparticle, leading to a superior signal amplification effect. Since then, several LFIs with gold ion signal amplification have been developed^19–24^.

However, these systems exhibit several disadvantages. For example, the reagents used for signal amplification must be added manually, and there is a long lag-time (≥10 min) after the immunoassay, rendering the system unsuitable for POC diagnosis (time lag-related complexity is not easily managed by nonexperts while performing an immunoassay). To make the LFI more user-friendly, various fluid flow control methods (also known as delayed-release of reagent-contained solution for enhancement methods) have been employed, including the use of wax filler^25^, dissolvable bridge^26^, sugar barrier^27^, delayed-release membrane^28^, valving system^29^, pressed-physical barrier^30,31^, pullulan film^32^, water-soluble nanofibers^33^, wax barrier^34^, and wax-printed channels^35^. Nevertheless, the complexity of these methods limits their application, reproducibility, and large-scale production. Thus, a highly sensitive LFI method based on a simple structure to delay the release of the signal-amplification solution, without long lag-time procedures, is warranted.

We previously reported the development of a trap LFI sensor (trapLFI) for sensitively and quantitatively detecting cortisol in saliva^36^. The trapLFI sensor has deletion and detection zones loaded with target-protein conjugate and an anti-mouse IgG antibody, respectively. By lowering the amount of AuNP-antibody conjugate, conjugates that do not react with the free analytes are completely "trapped" in the detection zone, whereas only the AuNP-antibody conjugate associated with the free analyte can escape from the deletion zone and react with immobilized secondary antibody on the detection zone, leading to a signal display. Although the platform showed high sensitivity and selectivity for detecting cortisol via ratio calculation of the quantified deletion and detection zones, an enhancement step with a long lag-time procedure was employed to amplify the signal. Thus, a new platform that can perform an immunoassay and a signal amplification in one-step could be highly advantageous for POC cortisol detection.

Herein, we report the development of an advanced trapLFI sensor, the “*α*-trapLFI” sensor. In this sensor, automatic signal amplification is achieved using water-soluble tape, and long lag-time procedures are not required for signal amplification. Polyvinyl alcohol (PVA) tape, which is commercially available, has excellent adhesive properties and is water-soluble. This tape is placed between the signal amplification pad and membrane where the immune reaction occurs. When the PVA tape is exposed to water, it dissolves and the structure collapses, allowing ions to pass through. This process takes approximately 5 min, facilitating the time delay between the immune reaction and signal amplification reaction. These properties enabled signal amplification in our sensor system. Although the sample solution and signal amplification solution were applied consecutively, the PVA tape between the conjugate pad and membrane for the immune reaction delayed the flow of signal-amplification solution through the membrane of the LFI. As a consequence, signal amplification only occurred after the immunoassay had proceeded for a set amount of time. This method can "simultaneously and automatically" induce signal amplification, which allows for target detection with high sensitivity in a user-friendly manner. The PVA tape-insulated LFI was successfully applied to detect cortisol in bodily fluids, such as saliva, serum, and urine, with high sensitivities. Our novel *α*-trapLFI provides a new benchmark for simple, rapid, and portable POC diagnosis of stress disorders.

## Results and discussion

### Principle of α-trapLFI sensor

**Scheme 1:**
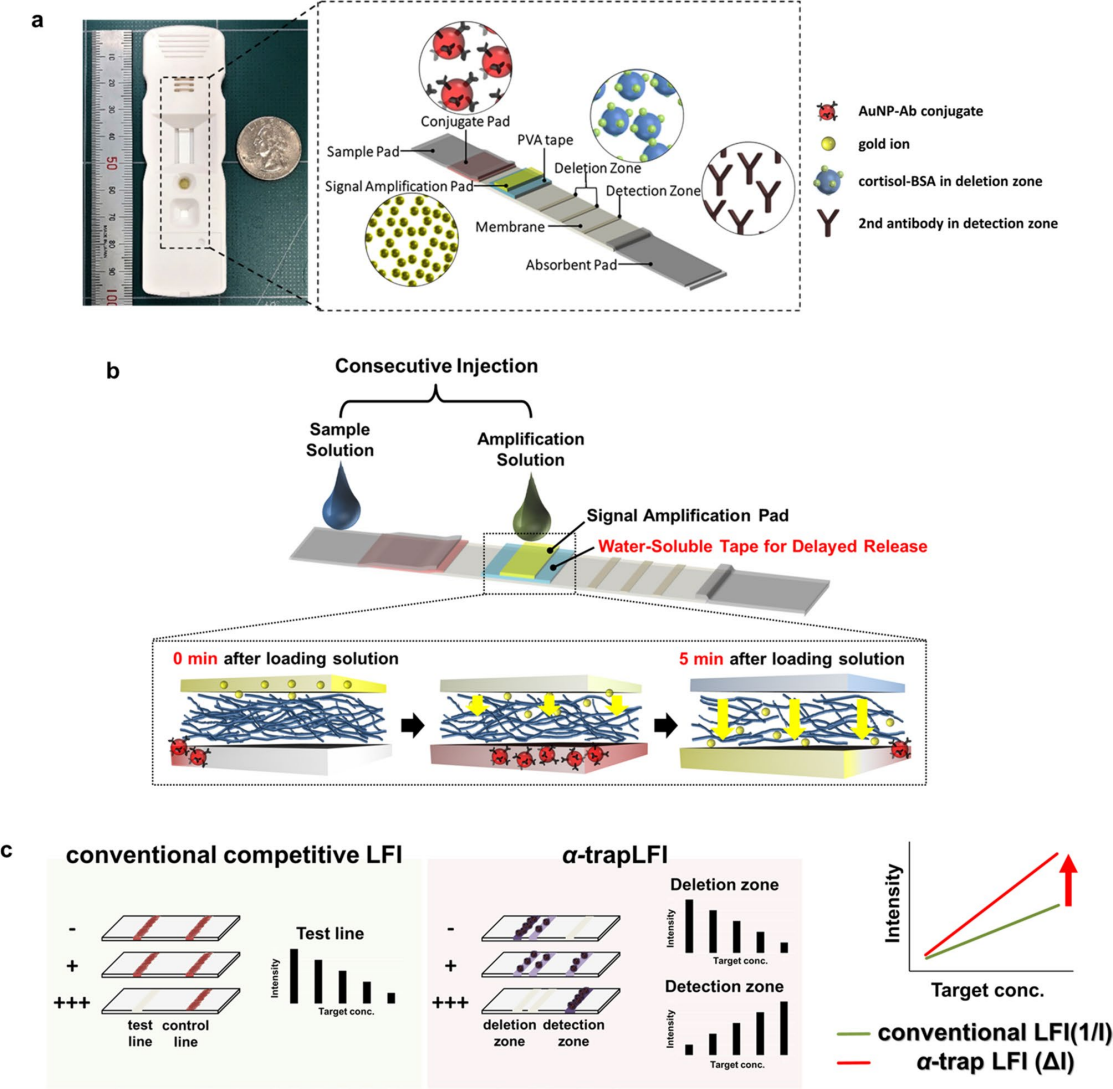
Schematic illustration of the new *α*-trapLFI sensor. (**a**) An digital image and schematic illustration of *α*-trapLFI sensor which consists of sample pad, conjugate pad, signal amplification pad, PVA tape, absorbent pad, and detection and deletion zone on membrane. (**b**) Signal amplification pad is placed on water-soluble polyvinyl alcohol (PVA) tape. After injecting sample and signal amplification solutions consecutively into the sensor, the detection antibody-gold nanoparticles conjugate passes first (the release of the gold ions is delayed by the PVA tape). After the conjugate passes, the PVA tape dissolves allowing the diffusion of ions, thus increasing the signal of the conjugate. (**c**) Comparison of conventional competitive LFI and *α*-trap LFI; although conventional LFI sensors can detect the target at high concentrations, the *α*-trapLFI sensor can detect the target at both low and high concentrations. This is due to two reasons: i) simultaneous and automatic signal amplification and ii) the ratiometric calculation with deletion and detection zones.

The proposed *α*-trapLFI sensor for cortisol analysis is shown schematically in Scheme 1. The sensor consists of a sample pad, conjugate pad, signal amplification pad, PVA tape, absorbent pad, and detection/deletion zones on the membrane (Scheme 1a). As the devised method involves the enlargement of AuNPs for signal amplification (Scheme S1), a signal amplification pad loaded with gold ions for signal amplification is placed on the PVA tape to induce a delayed-release effect. To investigate that the signal intensity is promoted by the enlargement of AuNPs growth, different concentration of AuNP conjugate applied strips were observed before and after introducing signal amplification solution. Before introducing the signal amplification solution, the signal can be detected from only 0.1X and 1X AuNP conjugate applied strips; however, after introducing the signal amplification solution, the signal can be detected from all strips (Fig. S1a and Fig. S1b).

When the sample and signal amplification solutions were consecutively injected onto the sample and the signal amplification pads, respectively, at the strip (without a long lag-time between injections), the PVA tape delayed the release of the gold ions at the signal amplification pad, whereas the target molecule in the sample solution and AuNP–antibody conjugate were promptly started to move forward and reached the detection zone. After approximately 5 min of exposure to water (amplification solution), subsequent dissolution of the PVA tape allowed the gold ions to flow from the signal amplification pad to the membrane, resulting in automatic sequential signal amplification (Movie S1). It should be noted that, in terms of sensitivity, the *α*-trapLFI sensor takes not only advantages of simultaneous and automatic signal amplification, but also those of trapLFI which was previously reported by our group^36^. In the trapLFI, a test line of a conventional LFI was used as a deletion zone which could trap target-free conjugates, and thus, only target-bounded conjugates could escape from deletion zone and captured at detection zone. Such a detection strategy resulted in high sensitivity due to ratiometric calculation between deletion and detection zones, and enabled quantitative and signal-on detection towards the target molecules. Therefore, unlike conventional competitive LFI that is only detectable at a high concentration of target, *α*-trapLFI is detectable at both low and high concentrations of target due to automatic signal amplification and the trap strategy (Scheme 1c).

### Characteristics of α-trapLFI sensor

#### Structure of PVA tape before and after exposure to solutions

**Figure 1:**
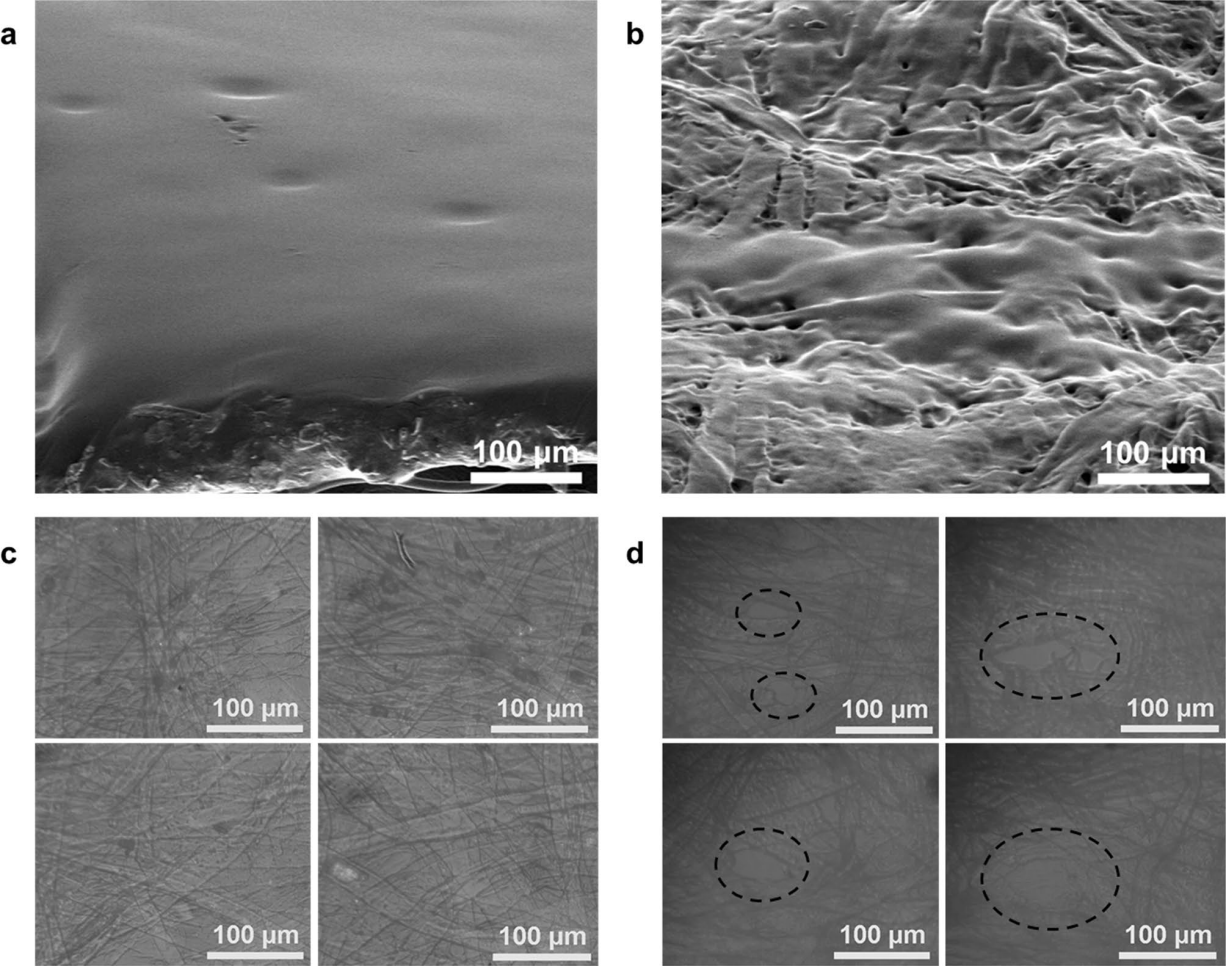
Images of polyvinyl alcohol (PVA) tape observed by (**a**,**b**) Scanning electron microscope and (**c**,**d**) optical microscopy. (**a**,**c**) Prior to loading solution; and (**b**,**d**) after loading solution. The surface of the PVA tape exposing to water is pitted, and collapsed structure and sizable holes were observed (dotted circles). The SEM images are 60° tilted views.

To investigate the structures of dry and wetted PVA tape, field emission scanning electron microscopy and optical microscopy were used. The surface of the PVA tape before exposure to water was polished (Fig. 1a). In contrast, the surface of the PVA tape was pitted after exposure to water (Fig. 1b). In addition, before exposure to water, interpenetrated fiber structure without distinct holes were observed (Fig. 1c); whereas a few of hole were observed after exposure to water (Fig. 1d). These results confirm that the PVA tape dissolved upon exposure to water, and that its rigid structure was collapsed and perturbed leading to a porous structure.

#### Delayed-release effect of PVA tape

**Figure 2:**
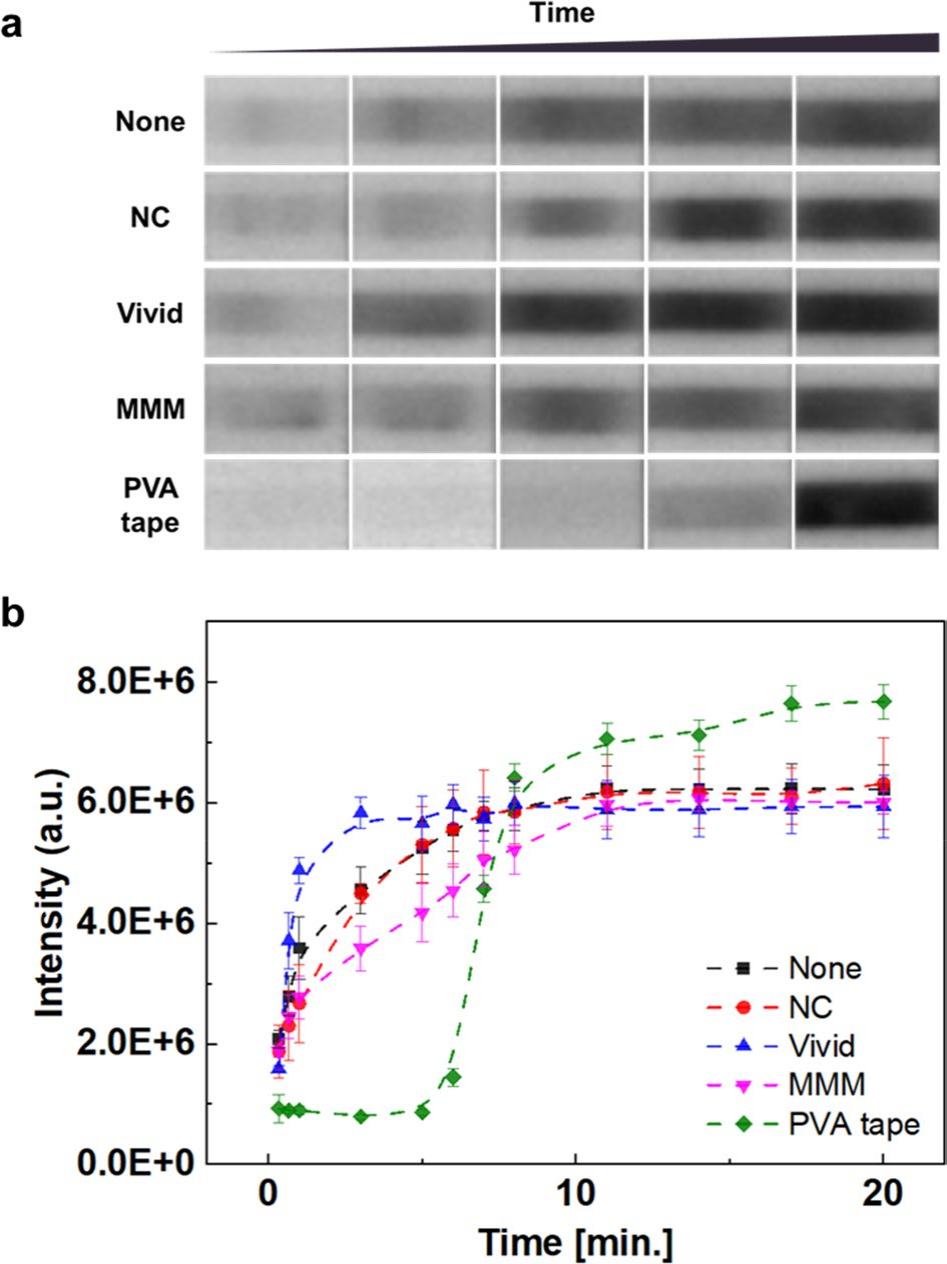
Delayed-release effect of the polyvinyl alcohol (PVA) tape compared with other membranes. (**a**) Time-dependent images obtained from assays. (**b**) Intensity plot. All images were obtained using a ChemiDoc XPS + imaging system (Bio-Rad), and the band intensities were measured using Image Lab software (version 6.1, https://www.bio-rad.com/en-uk/product/image-lab-software?source_wt=imagelabsoftware_surl&ID=KRE6P5E8Z). The error bars indicate the standard deviation from three independent experiments.

To confirm the delayed-release effect, the effect of PVA tape was compared to that of conventional membranes, including NC membranes, plasma separation (Vivid) membranes, and asymmetric (MMM) membranes. Thus, the various types of membranes were applied to the LFI sensor for comparative testing. Bare AuNP solution was immobilized on each membrane (1 µL∙cm^−1^), and a gold(III) ion-loaded signal amplification pads (8.2 µL∙3.8 mm^−2^) were applied onto each LFI. To initiate LFI, a sample solution (10% DMSO/PBS (v/v)) and signal amplification solution were consecutively loaded at the sample pad and he signal amplification pad, respectively. In the case of using a bare membrane, NC membrane, Vivid membrane, and MMM membrane, the signal amplification was occurred within 1 min. In contrast, a delayed-release effect was observed when PVA tape was used, and the signal amplification was started after approximately 5 min (Fig. 2 and Fig. S2). These results confirm that the PVA tape induced a superior delayed-release effect compared to the other membranes, and thus could be appropriately used as a signal amplification platform based on its delayed-release property.

The PVA tape was also compared to a general double-sided tape to further investigate the observed ion diffusion effect. For the comparison, the double-sided tape was placed between the signal amplification pad and membrane, and the same structure using PVA tape instead of the double-sided tape was prepared. The sample and amplification solutions were applied to the sample and signal amplification pads, respectively. No signal appeared over time on the strip with the general double-sided tape, whereas the signal intensity increased on the strip with the PVA tape at approximately 5–7 min after loading the sample and amplification solutions (Fig. S3a). A comparison of the resulting signal intensities (Fig. S3b) confirmed that only the PVA tape permitted ion diffusion.

The ion-diffusion permissible property of PVA tape may be attributed to its water-solubility. To confirm this, the general double-sided and PVA tapes (size 3.8 × 8.0 mm^2^) were exposed to water, and the weight was measured at different time points. Each tape was completely dried in an oven to reduce deviation, and the weight was measured using an ultra-microbalance. The general double-sided tape showed no change in weight after exposure to water. Whereas the weight of the PVA tape decreased with the time of exposure to water, decreasing by 19.5% after 5 min (Fig. S3c). Thus, the ion diffusion permissible property of PVA tape was attributed to water dissolution.

#### Automatic signal amplification using PVA tape

To verify an automatic signal amplification using PVA tape, the signal intensities of a conventional LFI and PVA tape-applied LFI were compared. Regarding the conventional LFI, anti-mouse IgG antibody were immobilized on a membrane (1 µL∙cm^−1^), and an AuNP–antibody conjugate (onefold) loaded pad was applied to the membrane. Regarding the PVA tape-applied LFI, a PVA tape (3.8 × 8.0 mm^2^) and a gold(III)-ion loaded pad (8.2 µL∙3.8 mm^−2^) were additionally applied to the membrane. The sample solution was loaded for conventional LFI, and the sample solution and signal amplification solution were consecutively loaded for PVA tape-applied LFI.

**Figure 3:**
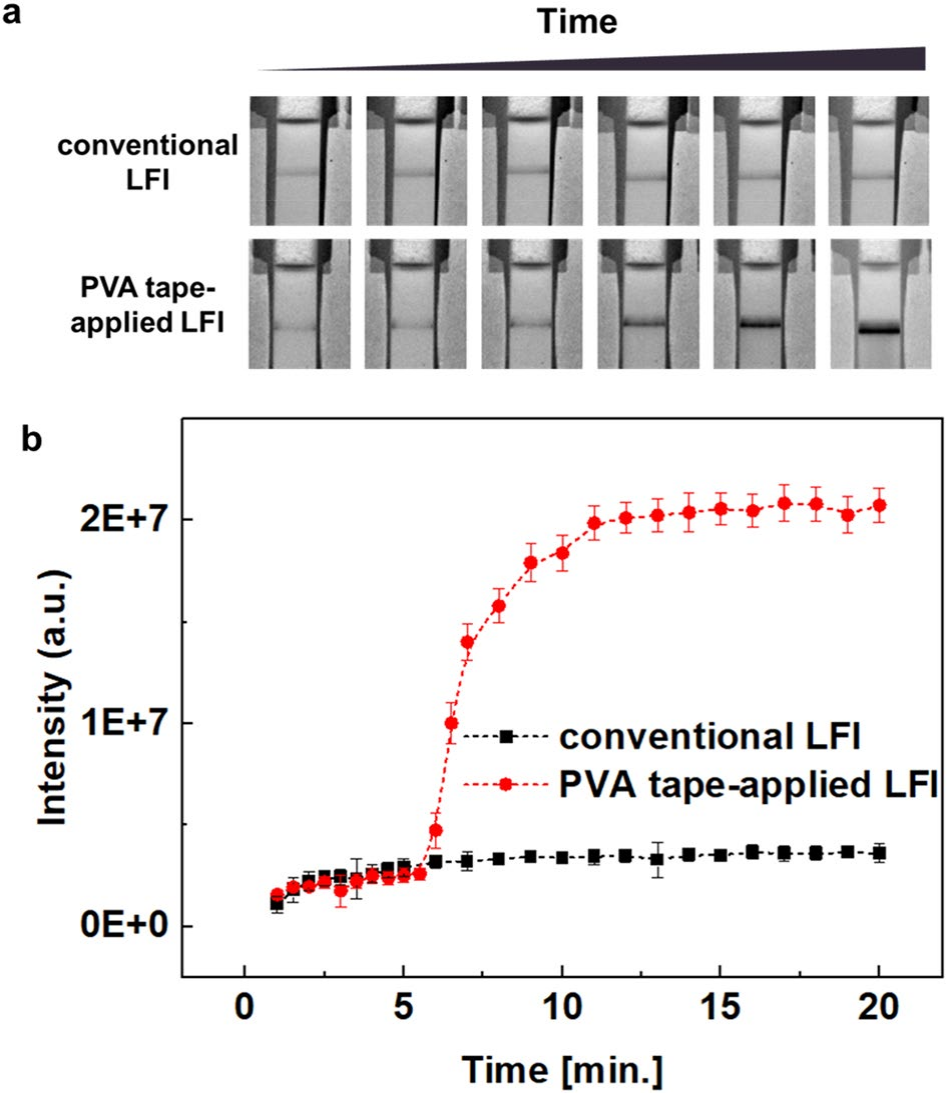
Comparison of signal intensities from a conventional lateral flow immunoassay (LFI) and a polyvinyl alcohol (PVA) tape-applied LFI. (**a**) Time-dependent images obtained from assays. (**b**) Intensity plot. All images were obtained using a ChemiDoc XPS + imaging system (Bio-Rad), and the band intensities were measured using Image Lab software (version 6.1, https://www.bio-rad.com/en-uk/product/image-lab-software?source_wt=imagelabsoftware_surl&ID=KRE6P5E8Z). Error bars indicate the standard deviation from three independent experiments.

Comparison of the signal intensities obtained from conventional LFI and PVA tape-applied LFI is shown in Fig. 3. In PVA tape-applied LFI, the signal was amplified approximately 5–6 min after sample and amplification solution injection, and a higher signal (compared with conventional LFI) was achieved (Fig. 3). As shown in Fig. 3b, the signal achieved from PVA tape-applied LFI was approximately 6.9-fold higher than that achieved from conventional LFI.

#### α-trapLFI sensor for detecting small molecules

In the previous report on the trapLFI sensor developed by our group^36^, we found that detection of cortisol with high sensitivity could be achieved by applying deletion and detection zones, rather than the test and control zones used in typical LFI. Moreover, because we used a small amount of AuNP–antibody conjugate, the conjugate not reacting with antigen could be effectively "trapped" at the deletion zone, minimizing the false-positive signal in the detection zone. However, the amount of conjugate could not be reduced further for the trapLFI sensor, as the signal needed to be detectable to measure its intensity. Herein, our previous trapLFI sensor was adopted as a platform to which the PVA tape-mediated delayed release method could be appended. In our modified sensor, the advanced trap-LFI (*α*-trapLFI), the advantages of the trapLFI sensor are combined with signal amplification by delayed release of gold ions to further increase sensitivity. As shown in Figure S4, although weak signal was detected in a conventional LFI using the lowest amount of AuNP–antibody conjugate (Fig. S4 left), a strong signal was detected when the *α*-trapLFI sensor was used due to signal amplification by delayed-release gold ions and a reducing agent (Fig. S4 right). In the *α*-trapLFI platform, the delayed-release effect induced by PVA tape was essential as shown in Fig. 2. The absence of PVA tape causes no signal because the gold ions were released before immunoreaction was occurred (Fig. S4 middle). Notably, the PVA tape was confirmed to have a negligible effect on the immunoreaction (Fig. S5).

**Figure 4:**
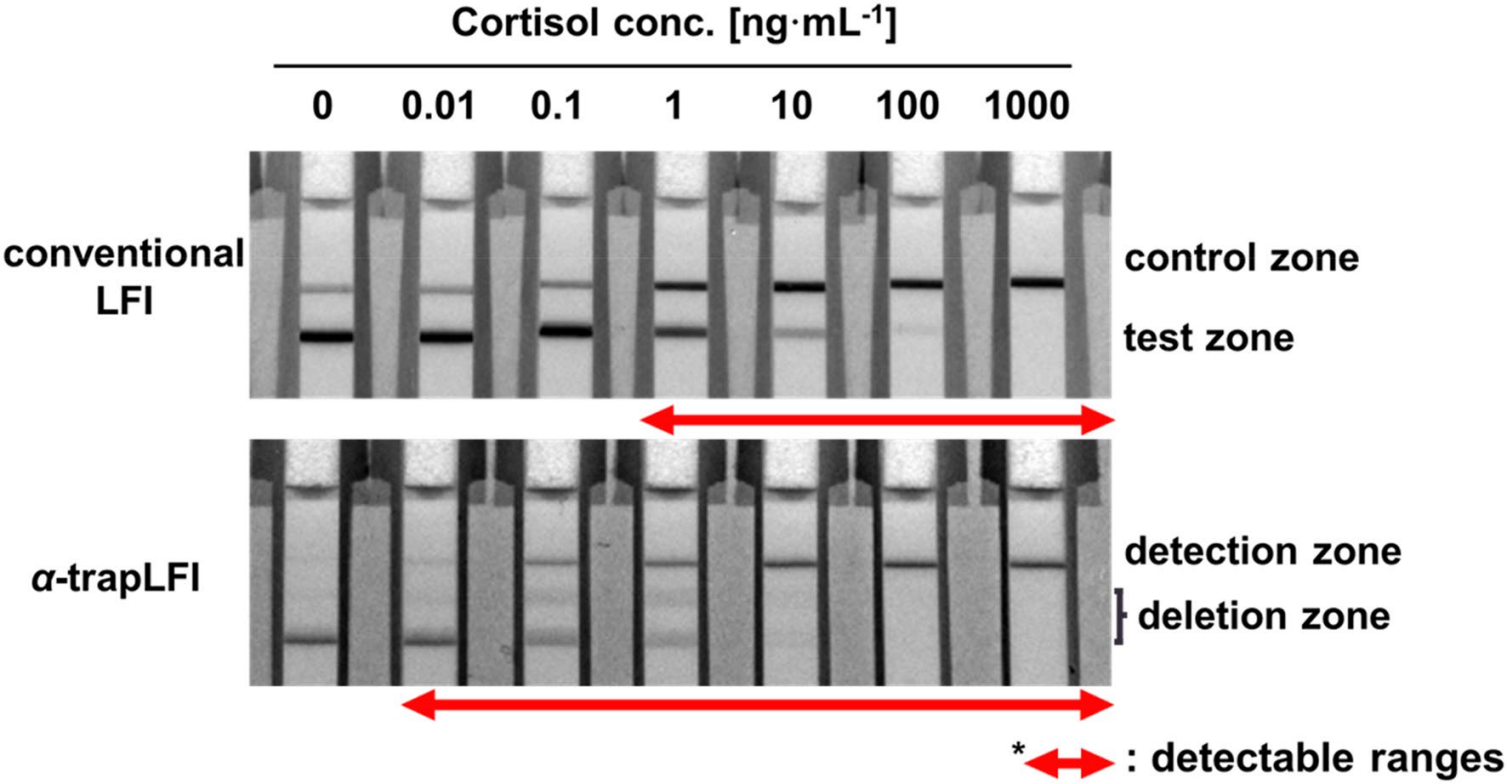
Sensitivity comparisons of the conventional lateral flow immunoassay (LFI) sensor and *α*-trapLFI sensor. All images were obtained using a ChemiDoc XPS + imaging system (Bio-Rad), and the band intensities were measured using Image Lab software (version 6.1, https://www.bio-rad.com/en-uk/product/image-lab-software?source_wt=imagelabsoftware_surl&ID=KRE6P5E8Z).

Comparison of the conventional LFI sensor and *α*-trapLFI sensor for cortisol analysis was also conducted to evaluate sensitivity (Fig. 4). The target-BSA conjugate and anti-mouse IgG antibody was immobilized on a membrane (1 µL∙cm^−1^), and then a conjugate pad loaded with the AuNP-antibody conjugate (onefold for conventional LFI; 0.001-fold for *α*-trapLFI) was applied to the membrane. For the *α*-trapLFI sensor, a signal amplification pad, PVA tape, and signal amplification solution were additionally applied, and the assay was carried out as described above. The results demonstrate that the conventional LFI shows the signal changes from about 1 ng/mL, whereas the *α*-trapLFI shows a different signal even at a lower concentration of cortisol. Thus, cortisol was detected with high sensitivity over a broad-detection range with the *α*-trapLFI sensor.

### Sensitivity and selectivity of the α-trapLFI sensor

**Figure 5:**
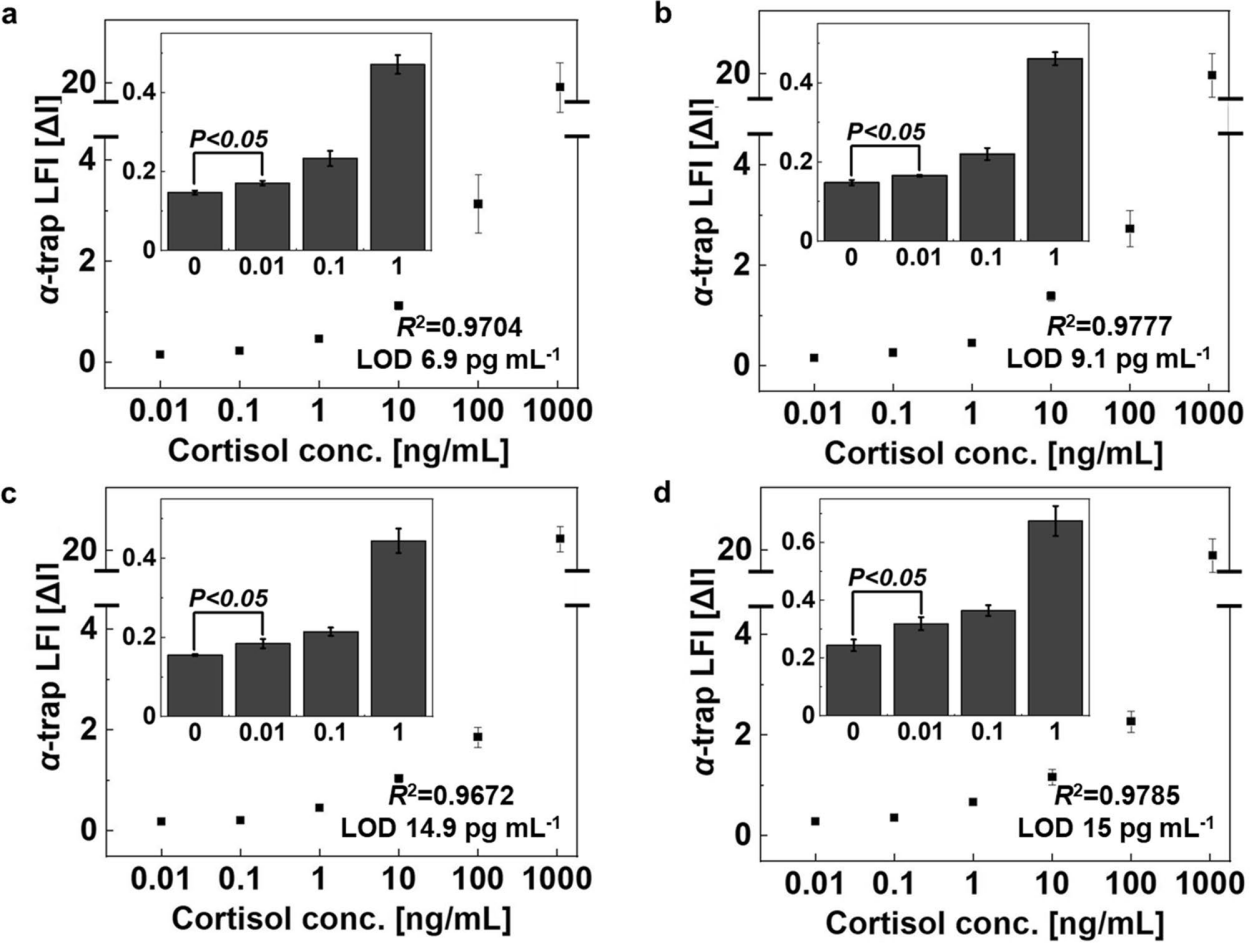
Sensitivity of *α*-trapLFI sensor for cortisol determination in human bodily fluids. Cortisol standard in (**a**) buffer, (**b**) saliva, (**c**) serum, and (**d**) urine. All images were obtained using a ChemiDoc XPS + imaging system (Bio-Rad), and the band intensities were measured using Image Lab software (version 6.1, https://www.bio-rad.com/en-uk/product/image-lab-software?source_wt=imagelabsoftware_surl&ID=KRE6P5E8Z). Error bars indicate the standard deviation from three independent experiments.

Our *α*-trapLFI sensor with simultaneous signal amplification accomplished by using the delayed release effect of PVA tape was successfully optimized. Next, cortisol was detected in buffer, human saliva, human serum, and human urine, to evaluate the performance of the *α*-trapLFI sensor. The sensitivity of the *α*-trapLFI sensor was tested by using cortisol standard solutions at various concentrations in DMSO, saliva, serum, and urine/buffer (10:90, v/v). The colorimetric signal intensities were determined at 15 min after consecutively adding the loading sample and signal amplification solutions, and the results were obtained by calculating the ratios of signals observed in the deletion and detection zones (ΔI (Delta I) = sum (deletion zones)/detection zone). As shown in Fig. 5 and Figure S6a, the *α*-trapLFI sensor was highly sensitive to cortisol in various human bodily fluids. The limit of detection (LOD) for the *α*-trapLFI was calculated as the sum of the blank signal plus 3 × the standard deviation of a control cortisol sample. LOD values for cortisol in DMSO, saliva, serum, and urine were 6.9, 9.1, 14.9, and 15 pg∙mL^−1^, respectively, and the signal intensity was linear from 0.01 to 100 ng∙mL^−1^ for all bodily fluids with an *R*^2^ of 0.9704, 0.9777, 0.9672, 0.9785 for buffer, saliva, serum and urine sample (Fig. S6b).

Table 1 shows a comparison of the characteristics of the *α*-trapLFI sensor with previously reported LFI. The *α*-trapLFI sensor demonstrated a better LOD with a broader detection range compared to the other sensors, and it could successfully detect cortisol in various human bodily fluids.

**Table 1:** Comparison of the specificities of *αlpha*-trapLFI with previously developed lateral flow immunoassay (LFI).

Ref	Limit of detection (LOD)	Assay time	Detection range	Enhancement method	Additional steps for signal amplification	Applied human fluids
^38^	0.3 ng∙mL^-1^	25 min	~ 60 ng∙mL^-1^	Enzyme enhancement	Addition of washing and enhancing solutions 10–15 min after sample solution injection	Saliva
^39^	0.1 ng∙mL^-1^	15 min	~ 100 ng∙mL^-1^	Gold ion enhancement	Addition of enhancing solutions 15 min after sample solution injection	Saliva
^40^	2.5 ng∙mL^-1^	15 min	~ 50 ng∙mL^-1^	-	-	Saliva
^36^	9.9 pg∙mL^-1^	15 min	~ 100 ng∙mL^-1^	Enzyme enhancement	Addition of enhancing solution 10 min after sample solution injection	Saliva
*α*-trapLFI	6.9 pg∙mL^-1^	10–15 min	~ 1000 ng∙mL^-1^	Gold ion enhancement	Addition of enhancing solution and sample solution consecutively	Saliva, serum, urine

**Figure 6:**
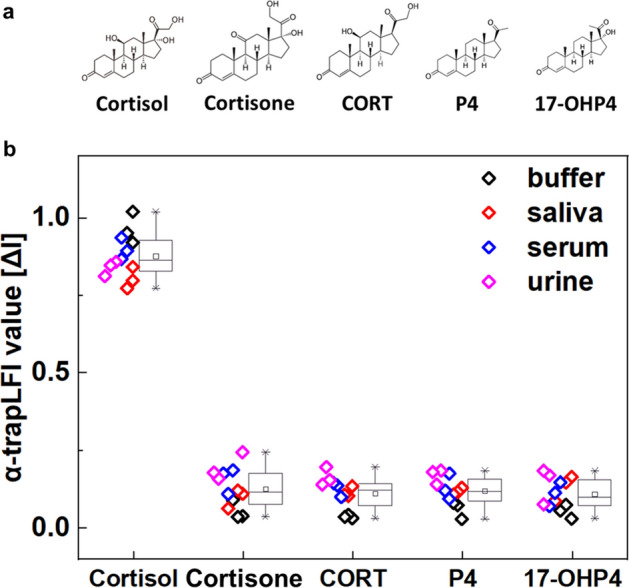
Cross-reactivity of *α*-trapLFI sensor in human bodily fluids. **a** Structure of cortisol and other steroid hormones. **b** Selectivity results comparing cortisol and other steroid hormones. All images were obtained using a ChemiDoc XPS + imaging system (Bio-Rad), and the band intensities were measured using Image Lab software (version 6.1, https://www.bio-rad.com/en-uk/product/image-lab-software?source_wt=imagelabsoftware_surl&ID=KRE6P5E8Z). Error bars indicate the standard deviation from three independent experiments.

Cross-reactivity of the *α*-trapLFI sensor was evaluated by exposure to other steroid hormones (Fig. 6a), including cortisone, corticosterone (CORT), progesterone (P4), and 17α-hydroxyprogesterone (17-OHP4), at 10 ng∙mL^-1^. The results of these assays are shown in Fig. 6b and Figure S7. The cortisol specificity of the *α*-trapLFI sensor was high, and no cross-reactivity with the other steroid hormones was observed. Thus, the *α*-trapLFI sensor can be used as a rapid and simple detection method for cortisol diagnosis.

### Analysis of cortisol in human saliva

**Figure 7:**
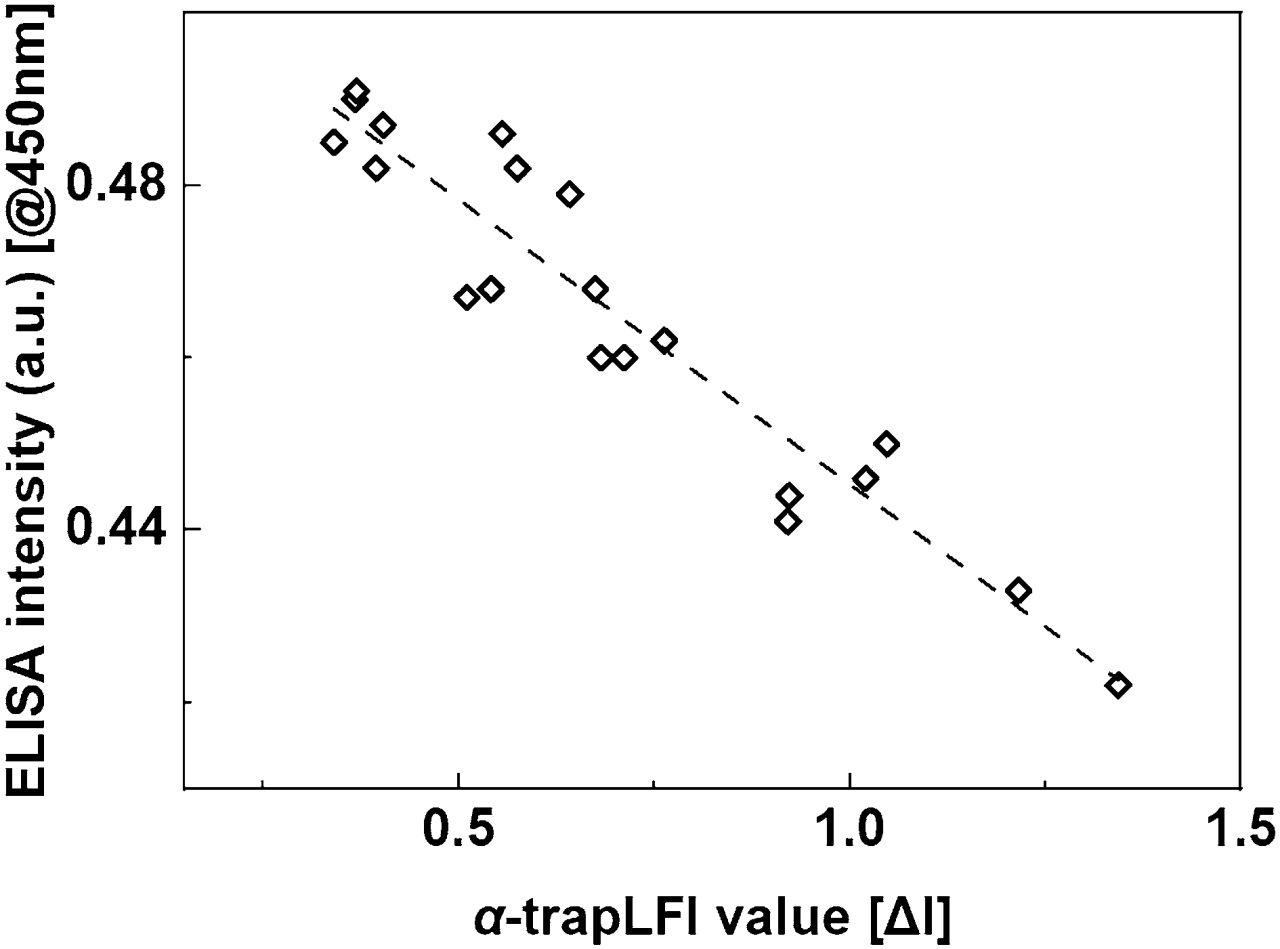
Human salivary cortisol level measured using conventional ELISA and *α*-trapLFI sensor. All images were obtained using a ChemiDoc XPS + imaging system (Bio-Rad), and the band intensities were measured using Image Lab software (version 6.1, https://www.bio-rad.com/en-uk/product/image-lab-software?source_wt=imagelabsoftware_surl&ID=KRE6P5E8Z).

Twenty human saliva samples were tested using the *α*-trapLFI sensor. The values obtained from the saliva test samples analyzed on the *α*-trapLFI sensor were compared with those analyzed by ELISA which is widely used as a gold standard method (Fig. 7), revealing the following correlation: *y* =  − 0.0661*x* + 0.5114 (*R*^2^ = 0.90). The above correlation indicated reliable agreement with the ELISA results. Hence, the *α*-trapLFI sensor can be used as a highly reliable sensor platform for detecting cortisol in real saliva samples. Together, our results support that our *α*-trapLFI sensor is a simple and practical platform for cortisol detection in human bodily fluids with high sensitivity.

## Conclusion

In this study, we developed an *α*-trapLFI sensor for simultaneous signal amplification without long lag-time procedures, which takes advantages of the trapLFI sensor previously reported by our group. The application of PVA tape, which exhibits delayed release effect, enable to amplify the signal without additional long lag-time steps. In addition, the performance of the *α*-trapLFI sensor was superior because the conjugates that did not react with the target were 'trapped', resulting in no signal in the detection zones when there was no target.; this is an inherent advantage of a trapLFI sensor. The performance of proposed sensor was verified using human bodily fluids including saliva, serum, and urine, and the correlation of the assay with the ELISA results was *R*^2^ = 0.90 in the real saliva samples. Particularly, the PVA tape used for simultaneous signal amplification is inexpensive and easy to procure and apply to signal amplification sensors; it also has a simple structure. Therefore, it can be easily applied to all LFI platforms to detect not only small molecules, but also various other targets.

## Methods

### Chemicals and reagents

Free cortisol, bovine serum albumin (BSA), cortisol-BSA, and surfactant 10G (S10G) were purchased from Fitzgerald Industries International (Acton, MA, USA). The anti-cortisol antibody was purchased from Abcam (Cambridge, UK), and the nitrocellulose (NC) membrane (FF80HP) was purchased from GE Healthcare (Chicago, IL, USA). Salivette Cortisol, which was used to collect saliva samples, was purchased from Sarstedt (Numbrecht, Germany). The absorbent pad (Grade222), conjugate pad (6613), signal amplification pad (8964), and sample pads (Grade222) were purchased from Ahlstrom-Munksjö (Helsinki, Finland). The NC membrane without backing (HF180UBXSS) was purchased from EMD Millipore (Burlington, MA, USA), and the Vivid plasma separation-GX membrane (T9EXPPA0200S00X) and asymmetric membrane—MMM0.45 (T9PA045W000M) were purchased from Pall Corporation (Port Washington, NY, USA). PVA tape (126,057) and a general double-sided tape (DTS-310) were purchased from Buho, Inc. (Daegu, Korea) and DUCKSUNG HITECH, Inc. (Gwangju, Korea), respectively. Gold nanoparticles (EM.GC15) and the TMB substrate reagent set (555,214) were purchased from BB International (Cardiff, UK) and BD Biosciences (San Jose, CA, USA), respectively. The ELISA microplate (655,061) was purchased from Greiner Bio-One International GmbH (Mühlkreis, Austria). Phosphate-buffered saline (PBS), sodium phosphate (monobasic), and sodium phosphate (dibasic) were purchased from Biosesang Co. (Sungnam, Korea). Neo protein saver (NPS-301) was purchased from TOYOBO Co., Ltd. (Osaka, Japan). An anti-mouse IgG antibody produced in goat, boric acid, sodium tetraborate decahydrate, trisodium citrate dihydrate, citric acid, polyvinylpyrrolidone (10 K) (PVP 10 K), D-( +)-trehalose dehydrate, Triton X-100, Tween 20, dimethyl sulfoxide (DMSO), gold(III) chloride trihydrate, hydroxylamine hydrochloride, sulfuric acid, cortisone, corticosterone (CORT), progesterone (P4), 17α-hydroxyprogesterone (17-OHP4), human serum, and all other chemicals were purchased from Sigma-Aldrich (St. Louis, MO, USA). Normal human saliva fluid and normal human urine fluid were purchased from MyBioSource (San Diego, CA, USA). Water was purified using a lab water purification system (PURELAB Option-Q, ELGA LabWater Ltd., Lane End, UK).

### Characterization of PVA tape

The structure of PVA tape was characterized by field emission scanning electron microscopy and light microscopy. The PVA tape characteristics were also compared to a general double-sided tape to determine its delayed-release effect. AuNP colloid (15 nm in diameter, 1 × AuNP, *λ*_max_ O.D. = 1.0) was immobilized on an NC membrane (1 μL·cm^-1^), and then gold(III) chloride trihydrate (0.1 M) was applied on the signal amplification pad. The general double-sided and PVA tapes were attached beneath the signal amplification pad. The sample solution, 10% (v/v) DMSO/PBS, and the signal amplification solution, 10 mM hydroxylamine in 10 mM citrate (pH 3.8), were applied onto the sample and signal amplification pads, respectively. The weight of the PVA tape was measured at five time points after loading the buffer. The PVA and general double-sided tapes (size, 3.8 × 8.0 mm^2^) were placed in an e-tube containing citrate buffer (10 mM, pH 3.8) and vigorously vortexed. After 15 min, the tapes were dried completely, and their weights were measured using an ultra-microbalance.

Effective delayed release from the PVA tape was confirmed as described above with different membranes. To determine whether the PVA tape impacts the immunoassay, anti-mouse IgG antibodies (1 mg·mL^-1^) were immobilized on an NC membrane (1 μL·cm^-1^), and then AuNP–antibody conjugate (three-fold) was applied on a conjugate pad. The PVA tape was attached beneath the signal amplification pad, and the sample and signal amplification solutions were applied to the sample and signal amplification pads, respectively.

### Preparation of AuNP conjugate

The anti-cortisol antibody (5 μL, 2 mg·mL^-1^) was added to a mixture of 1 mL of AuNP colloid (15 nm in diameter, 1 × AuNP, *λ*_max_ O.D. = 1.0) and 100 μL of 0.1 M borate buffer (pH 8.5). After incubation for 30 min at 21 °C, 10 µL of protein saver (100 mg·mL^-1^ in PBS) was added to the mixture to block non-specific binding sites on the AuNP surface. After incubation for 1 h at 21 °C, the mixture was centrifuged at 15 602 × *g* for 15 min at 10 °C. The supernatant was discarded and the AuNP conjugate was resuspended in 1 mL of 10 mM borate buffer (pH 8.5), and the AuNP conjugate was concentrated by tenfold and stored in storage buffer composed of 5% (w/v) D-( +)-trehalose, 0.5% (w/v) Protein saver, 0.2% (v/v) Tween 20, and 1% (v/v) Triton™ X-100 in PBS, at 4 °C until use.

### Preparation of α-trapLFI sensor using PVA tape

To prepare an *α*-trapLFI sensor, 250 μg·mL^-1^ cortisol–BSA and 1 mg·mL^-1^ anti-mouse IgG antibody were immobilized on an NC membrane (1 μL·cm^-1^) to create two deletion zones and one detection zone, respectively; the distance between each zone was approximately 2.5 mm. An absorbent pad was attached on top of the membrane with a 2-mm overlap. The AuNP conjugate was diluted by 1000-fold with a solution containing 1% (w/v) PVP 10 K and 0.5% (w/v) S10G in PBS, and 3.61 μL of diluted AuNP conjugate was applied to the conjugate pad. Next, 8.2 μL of solution containing 0.1 M gold(III) chloride trihydrate in 10 mM phosphate buffer (pH 7.4) was applied to the signal amplification pad.

The AuNP conjugate-loaded conjugate pad was attached to the bottom of the prepared strip, and a sample pad was placed underneath it to load the sample. The PVA tape was attached to the membrane at 2-mm above the conjugate pad, and a gold(III) chloride trihydrate-loaded signal amplification pad was placed in the middle of the PVA tape. The assembled membrane was cut into 3.8-mm wide strips and stored in a humidity-controlled chamber (21 °C; 23% relative humidity) until use. Before use, the strip was placed in a plastic case provided by Infopia Co., Ltd. (Anyang, Gyeonggi, Korea).

### Cortisol analysis in buffer, saliva, serum, and urine samples

Cortisol solutions at various concentrations in DMSO, human saliva, serum, and urine were diluted tenfold in 1 × PBS buffer (10% concentration). The signal amplification solutions were prepared at 10 mM in citrate buffer (pH 3.8). Subsequently, 100 μL of the prepared sample solution (10% (v/v) DMSO/PBS) and 20 μL of the signal amplification solution (10 mM hydroxylamine in 10 mM citrate (pH 3.8)) were applied to the sample and signal amplification pads of the strip, respectively. The strip was then incubated at 21 °C for 20 min. In addition, several other steroid hormones were also analyzed with same methods described above. All images were obtained using a ChemiDoc XPS + imaging system (Bio-Rad, Hercules, CA, USA), and the band intensities were measured using Image Lab software (version 6.1, https://www.bio-rad.com/en-uk/product/image-lab-software?source_wt=imagelabsoftware_surl&ID=KRE6P5E8Z). After measuring the intensities of each strip, the value of ΔI (delta I) is introduced to increase sensitivity and quantitative results. The limit of detection was calculated with the mean value and the standard deviation (mean + 3SD).

### Collection of saliva samples

Human saliva samples were obtained from 20 volunteers from the Gwangju Institute of Science and Technology (GIST) in Gwangju, Korea. We collected samples of saliva from healthy individuals between 08:00 and 09:00 using a saliva collecting kit. The participants were asked to refrain from eating, drinking, and performing oral hygiene procedures until the morning of saliva collection. All participants provided informed consent to participate in the study. The study was approved by GIST (Approval number: 20190510-BR-45-08-02). The collected saliva samples were centrifuged at 1274×*g* for 5 min to remove cell debris and other proteins, and the collected saliva samples were immediately used for testing. All experiments were performed in accordance with relevant guidelines and regulations.

### Analysis of cortisol in human saliva samples

The results obtained using the sensor platform were compared to those obtained with an enzyme-linked immunosorbent assay (ELISA). Briefly, ELISA was performed in 96-well microplates. The anti-cortisol antibody (100 μL, 4 μg·mL^-1^) was added to each well, and the plates were incubated at 4 °C overnight. After removing unbound antibodies by washing the wells with PBS-Tween (137 mM NaCl, 0.05% (v/v) Tween 20, pH 7.4), the wells were blocked with 200 μL of BSA solution (0.5 mg·mL^-1^ in PBS) at 21 °C for 2 h. Next, individual wells were incubated with 100 μL of the diluted saliva samples and cortisol-HRP conjugate (1 μg·mL^-1^) mixture at 21 °C for 5 h. After removing all unbound proteins, the samples were incubated with TMB substrate solution (50 μL) in the dark at 21 °C for 10 min. Finally, sulfuric acid (50 μL, 2 N) was added to immediately stop the enzyme reaction. The absorbance of the solution was determined at 450 nm using a spectrometry system (Cytation 5; BioTek Instruments, Winooski, VT, USA).

### Instruments

A microcentrifuge (smart R17 plus; Hanil Scientific Inc., Gimpo, Korea) was used to separate the AuNP conjugate, and a multi-purpose centrifuge (1580R; LABOGENE Co., Ltd., Lillerød, Denmark) was used to collect saliva samples. A drying oven (KO-100; LK Lab Co., Namyangju, Korea) was used to dry the membranes and all pads following antibody and sample loading. A dispenser (DCI100; Zeta Co., Gunpo, Korea) was used to immobilize samples on a membrane, and a cutting device (TBC-50Ts; Taewoo Co., Namyangju, Korea) was used to cut the membranes. A field emission scanning electron microscope (Hitachi S-4700, Tokyo, Japan) and a light microscope (Olympus BX43, Tokyo, Japan) were used to characterize the structure of PVA tape. An XPR ultra-microbalance (XPR6UD5; Mettler Toledo, Masstron, OH, USA) was used to weigh tapes. All signals from the strips were measured and analyzed with a ChemiDoc XPS + imaging system and Image lab software (6.1; Bio-Rad Laboratories), respectively. The ELISA results were evaluated using a cell imaging multi-mode reader (Cytation 5).

## Supplementary Information

Supplementary Information 1.

Supplementary Video 1.

## Data Availability

All data generated or analyzed during this study are included in this published article (and its Supplementary Information files).

## References

[1] Yehuda R, Teicher MH, Trestman RL, Levengood RA, Siever LJ (1996). Cortisol regulation in posttraumatic stress disorder and major depression: a chronobiological analysis. Biol. Psychiatry.

[2] Meewisse M-L, Reitsma JB, De Vries G-J, Gersons BP, Olff M (2007). Cortisol and post-traumatic stress disorder in adults: systematic review and meta-analysis. Br. J. Psychiatry.

[3] Karlamangla AS, Singer BH, McEwen BS, Rowe JW, Seeman TE (2002). Allostatic load as a predictor of functional decline: MacArthur studies of successful aging. J. Clin. Epidemiol..

[4] Tan T (2020). Association between high serum total cortisol concentrations and mortality from COVID-19. Lancet Diabetes Endocrinol..

[5] Posener JA, DeBattista C, Williams GH, Kreamer HC, Kalehzan M, Schatzberg AF (2000). 24-hour monitoring of cortisol and corticotropin secretion in psychotic and nonpsychotic major depression. Arch. Gen. Psychiatry.

[6] Posthuma-Trumpie GA, Korf J, van Amerongen A (2008). Lateral flow (immuno) assay: Its strengths, weaknesses, opportunities and threats. A literature survey. Anal. Bioanal. Chem..

[7] The immunoassay handbook: theory and applications of ligand binding, ELISA, and Related Techniques (ed. Wild, D.) 89–95 (Elsevier, 2013).

[8] Sajid M, Kawde A-N, Daud M (2015). Designs, formats and applications of lateral flow assay: A literature review. J. Saudi Chem. Soc..

[9] Bahadır EB, Sezgintürk MK (2016). Lateral flow assays: principles, designs and labels. Trends Analyt. Chem..

[10] Busa LSA, Maeki M, Ishida A, Tani H, Tokeshi M (2016). Simple and sensitive colorimetric assay system for horseradish peroxidase using microfluidic paper-based devices. Sens. Actuat. B Chem..

[11] Chen Y (2016). A dual-readout chemiluminescent-gold lateral flow test for multiplex and ultrasensitive detection of disease biomarkers in real samples. Nanoscale.

[12] Gao X (2016). An enzyme-amplified lateral flow strip biosensor for visual detection of microRNA-224. Talanta.

[13] Park S, Kim H, Paek SH, Hong JW, Kim YK (2008). Enzyme-linked immuno-strip biosensor to detect Escherichia coli O157: H7. Ultramicroscopy.

[14] Liu Y (2015). A highly sensitive and flexible magnetic nanoprobe labeled immunochromatographic assay platform for pathogen Vibrio parahaemolyticus. Int. J. Food Microbiol..

[15] Loynachan CN (2018). Platinum nanocatalyst amplification: Redefining the gold standard for lateral flow immunoassays with ultrabroad dynamic range. ACS Nano.

[16] Park JM (2015). Chemiluminescence lateral flow immunoassay based on Pt nanoparticle with peroxidase activity. Anal. Chim. Acta..

[17] Taranova NA, Berlina AN, Zherdev AV, Dzantiev BB (2015). 'Traffic light' immunochromatographic test based on multicolor quantum dots for the simultaneous detection of several antibiotics in milk. Biosens. Bioelectron..

[18] Yang W (2011). A colloidal gold probe-based silver enhancement immunochromatographic assay for the rapid detection of abrin-a. Biosens. Bioelectron..

[19] Fridley GE, Le H, Yager P (2014). Highly sensitive immunoassay based on controlled rehydration of patterned reagents in a 2-dimensional paper network. Anal. Chem..

[20] Fu E (2011). Enhanced sensitivity of lateral flow tests using a two-dimensional paper network format. Anal. Chem..

[21] Fu E (2012). Two-dimensional paper network format that enables simple multistep assays for use in low-resource settings in the context of malaria antigen detection. Anal. Chem..

[22] Razo, S. C. et al. Enlargement of gold nanoparticles for sensitive immunochromatographic diagnostics of potato brown rot. *Sensors (Basel)* **19,** 153 (2019). 10.3390/s19010153 PMC6338966 30621133

[23] Rodriguez MO, Covian LB, Garcia AC, Blanco-Lopez MC (2016). Silver and gold enhancement methods for lateral flow immunoassays. Talanta.

[24] Rohrman BA, Leautaud V, Molyneux E, Richards-Kortum RR (2012). A lateral flow assay for quantitative detection of amplified HIV-1 RNA. PLoS ONE.

[25] Fridley GE, Le HQ, Fu E, Yager P (2012). Controlled release of dry reagents in porous media for tunable temporal and spatial distribution upon rehydration. Lab. Chip..

[26] Houghtaling J, Liang T, Thiessen G, Fu E (2012). Dissolvable bridges for manipulating fluid volumes in paper networks. Anal. Chem..

[27] Lutz B (2013). Dissolvable fluidic time delays for programming multi-step assays in instrument-free paper diagnostics. Lab. Chip..

[28] Joung HA, Oh YK, Kim MG (2014). An automatic enzyme immunoassay based on a chemiluminescent lateral flow immunosensor. Biosens. Bioelect..

[29] Toley BJ (2015). A versatile valving toolkit for automating fluidic operations in paper microfluidic devices. Lab. Chip..

[30] Park J, Park J-K (2017). Pressed region integrated 3D paper-based microfluidic device that enables vertical flow multistep assays for the detection of C-reactive protein based on programmed reagent loading. Sens. Actuat. B Chem..

[31] Park J, Shin JH, Park JK (2016). Pressed paper-based dipstick for detection of foodborne pathogens with multistep reactions. Anal. Chem..

[32] Jahanshahi-Anbuhi S (2017). Automating multi-step paper-based assays using integrated layering of reagents. Lab. Chip..

[33] Kim W, Lee S, Jeon S (2018). Enhanced sensitivity of lateral flow immunoassays by using water-soluble nanofibers and silver-enhancement reactions. Sens. Actuat. B Chem..

[34] Ishii M (2018). Wax-assisted one-step enzyme-linked immunosorbent assay on lateral flow test devices. Anal. Sci..

[35] Preechakasedkit P, Siangproh W, Khongchareonporn N, Ngamrojanavanich N, Chailapakul O (2018). Development of an automated wax-printed paper-based lateral flow device for alpha-fetoprotein enzyme-linked immunosorbent assay. Biosens. Bioelectron..

[36] Oh HK, Kim JW, Kim JM, Kim MG (2018). High sensitive and broad-range detection of cortisol in human saliva using a trap lateral flow immunoassay (trapLFI) sensor. Analyst.

[37] Ma Z, Sui SF (2002). Naked-eye sensitive detection of immunoglubulin G by enlargement of Au nanoparticles in vitro. Angew. Chem. Int. Ed. Engl..

[38] Zangheri M (2015). A simple and compact smartphone accessory for quantitative chemiluminescence-based lateral flow immunoassay for salivary cortisol detection. Biosens. Bioelectron..

[39] Yang J-S, Shin J, Choi S, Jung H-I (2017). Smartphone Diagnostics Unit (SDU) for the assessment of human stress and inflammation level assisted by biomarker ink, fountain pen, and origami holder for strip biosensor. Sens. Actuat. B Chem..

[40] Rey E, Jain A, Abdullah S, Choudhury T, Erickson D (2018). Personalized stress monitoring: a smartphone-enabled system for quantification of salivary cortisol. Pers. Ubiquitous Comput..

