# Supplementary material for: Advanced trap lateral flow immunoassay sensor for the detection of cortisol in human bodily fluids
Source: Sci Rep. 2021 Nov 19;11:22580. doi: 10.1038/s41598-021-02084-7 (PMC8604903; doi:10.1038/s41598-021-02084-7)
Supplement: Supplementary file 1 — Supplementary Information 1. [file 41598_2021_2084_MOESM1_ESM.docx]

Supplementary Information


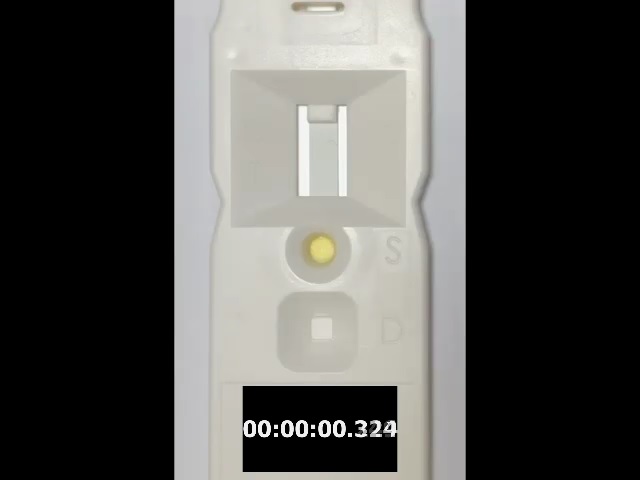


**Supplementary Movie S1**. Operation of *α*-trapLFI sensor.


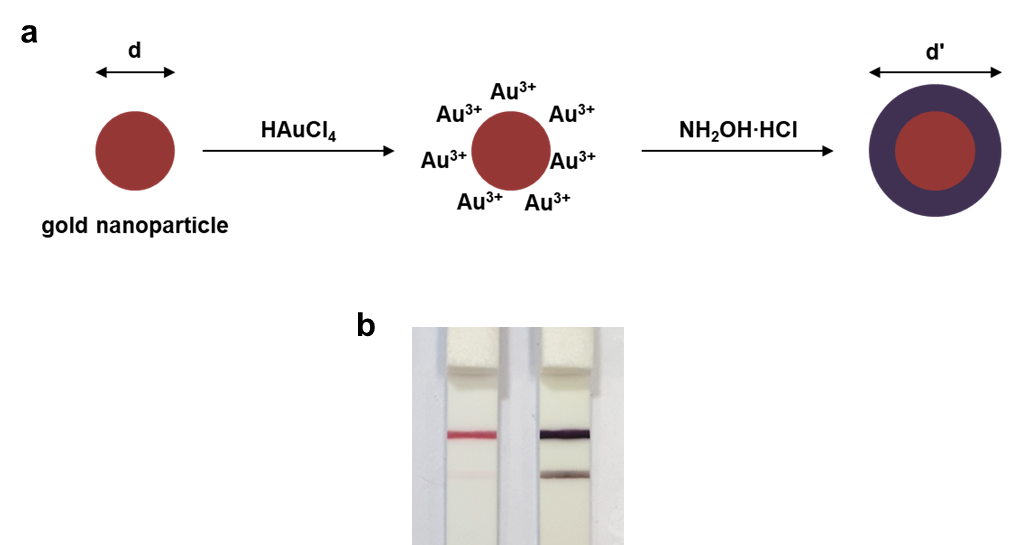


**Supplementary Scheme S1**. Schematic illustration of signal amplification used in this study for gold ion deposition. (a) The reaction of gold ions with a reducing agent. When gold ions are located near the gold nanoparticles and react with a reducing agent, they form gold nanoparticles that induce signal amplification, thus improving sensitivity. (b) A color image of strips (left) in a state performed only an immunoassay using gold nanoparticles conjugates; and (right) in a state performed a signal amplification by reducing gold ions after the immunoassay.

**
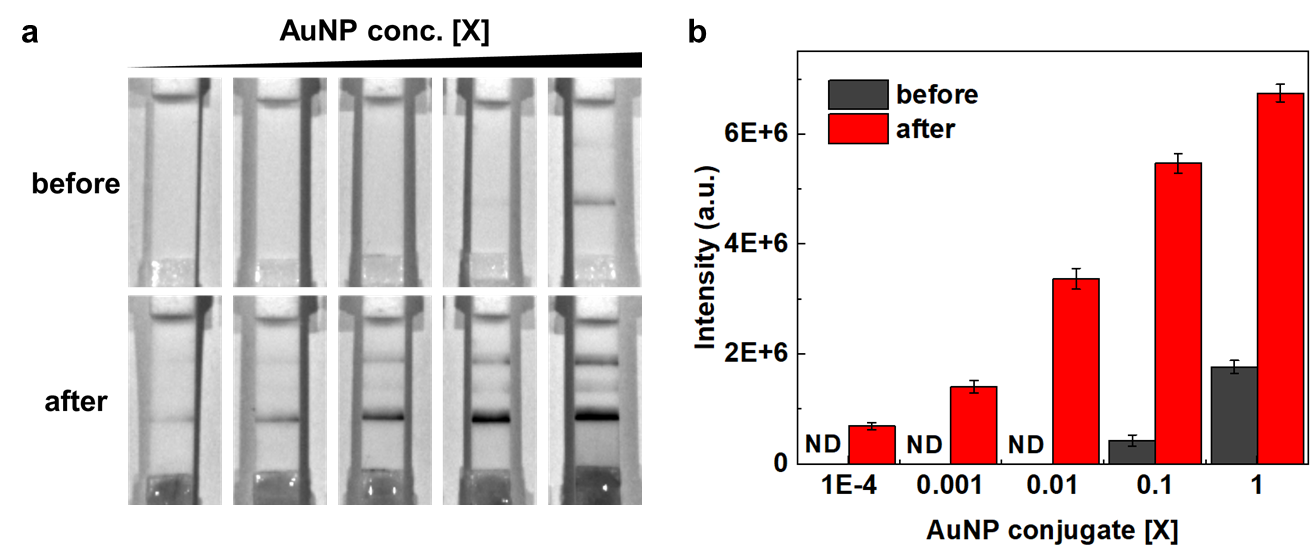
**

**Supplementary Fig. S1**. The signal intensity differences before and after the enlargement of AuNPs. (a) AuNPs concentration-dependent images obtained from assays. (b) Intensity plot. All images were obtained using a ChemiDoc XPS+ imaging system (Bio-Rad), and the band intensities were measured using Image Lab software (version 6.1, <https://www.bio-rad.com/en-uk/product/image-lab-software?source_wt=imagelabsoftware_surl&ID=KRE6P5E8Z>). Error bars indicate the standard deviation from three independent experiments.

**
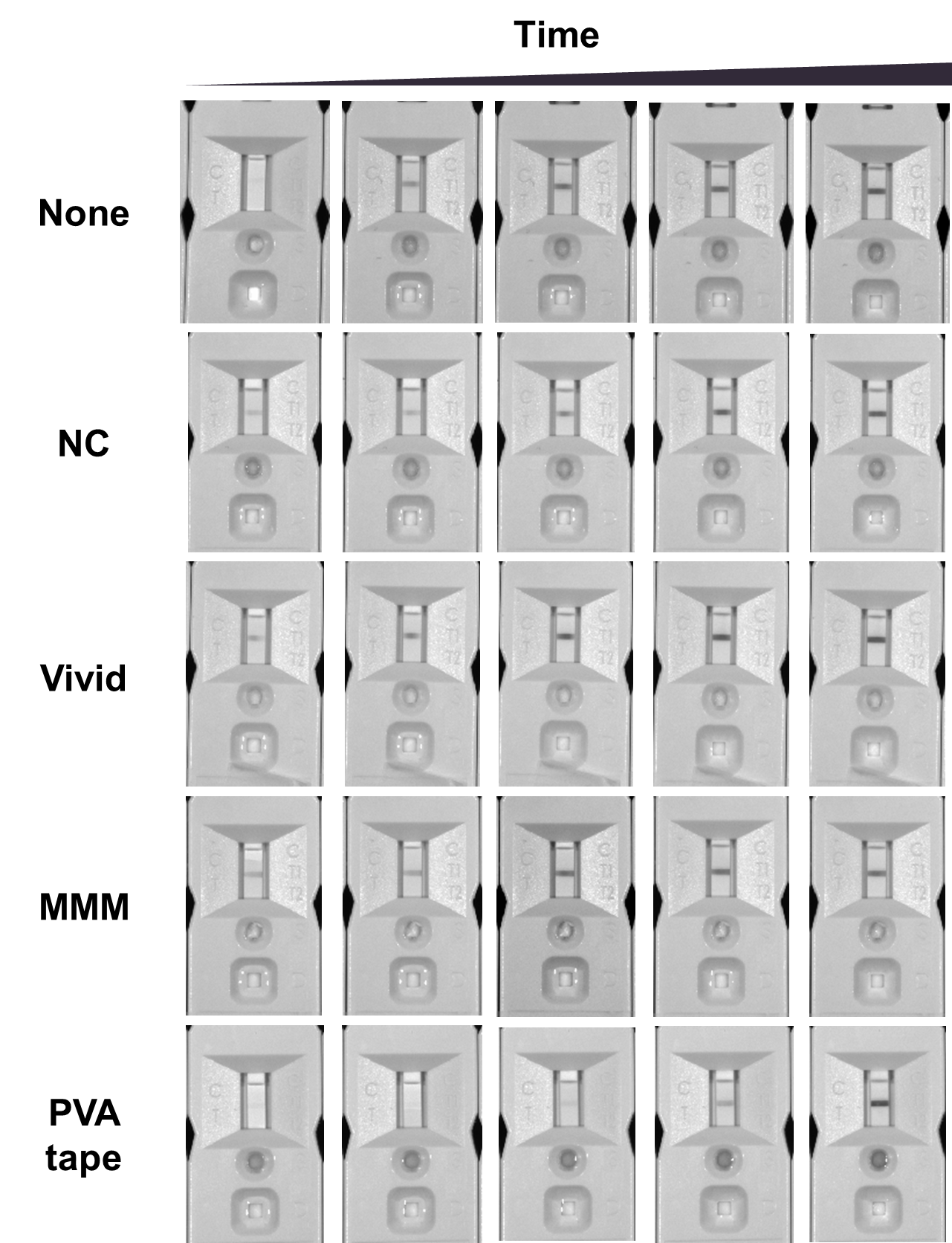
**

**Supplementary Fig. S2**. Images of each strip for confirming delayed-release effect with PVA tape and other membranes. All images were obtained using a ChemiDoc XPS+ imaging system (Bio-Rad).

**
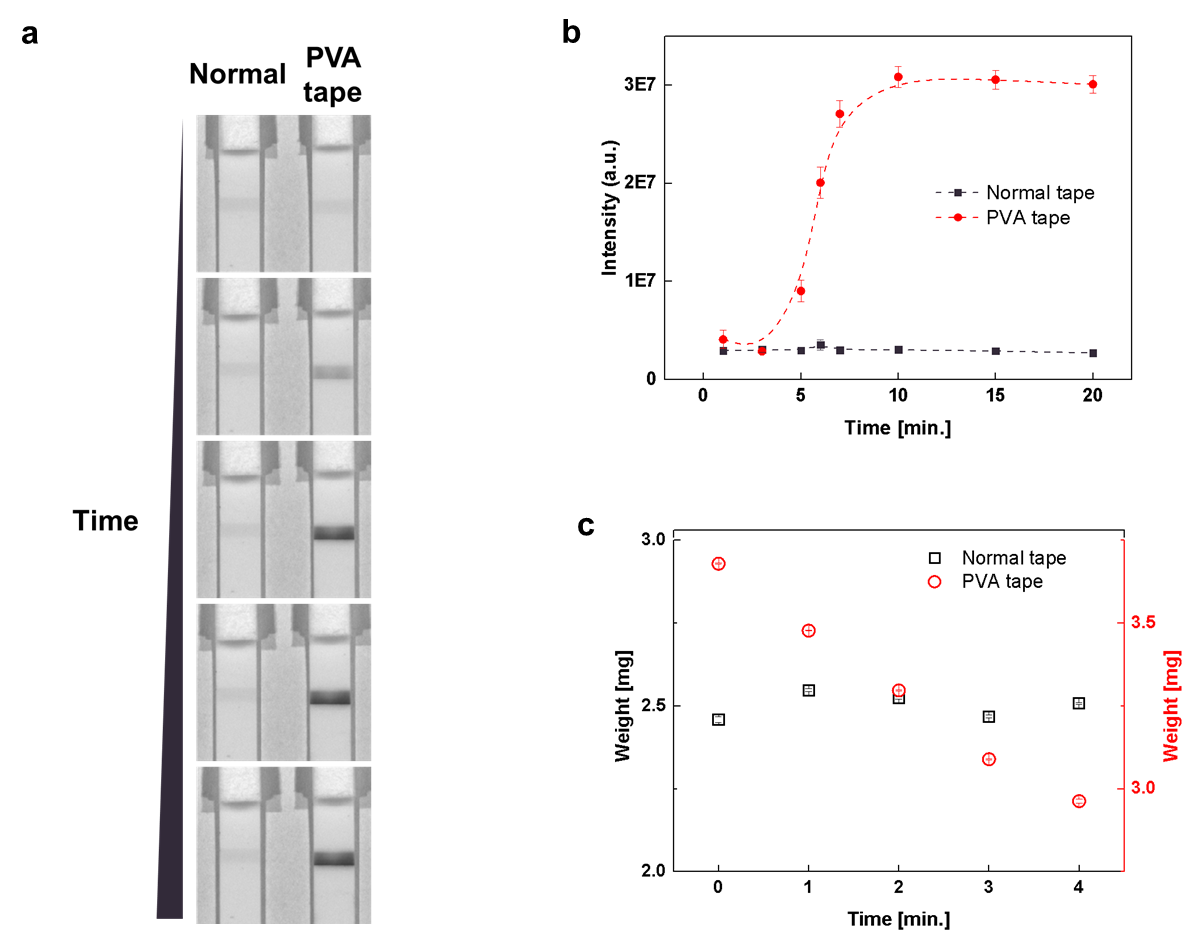
**

**Supplementary Fig. S3**. Delayed-release effect of PVA tape compared to general double-sided tape. (a, b) General double-sided tape was used to confirm the delayed-release effect of the PVA tape. (c) Weight of the general double-sided tape and PVA tape exposed to water. All images were obtained using a ChemiDoc XPS+ imaging system (Bio-Rad), and the band intensities were measured using Image Lab software (version 6.1, <https://www.bio-rad.com/en-uk/product/image-lab-software?source_wt=imagelabsoftware_surl&ID=KRE6P5E8Z>). Error bars indicate the standard deviation from three independent experiments. Error bars indicate the standard deviation from three independent experiments.


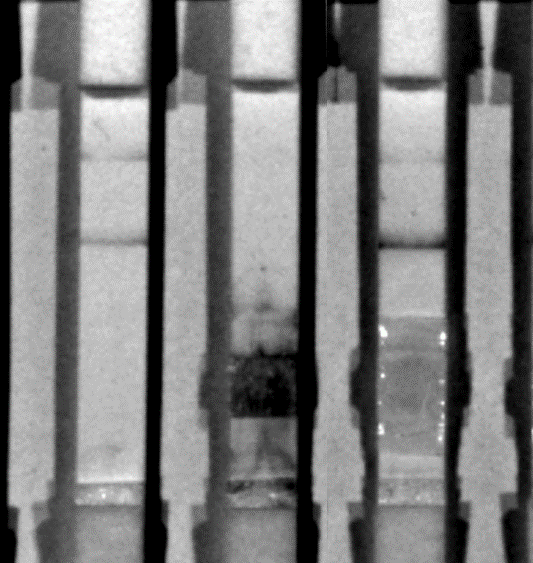


**Supplementary Fig. S4**. Comparison of conventional lateral flow immunoassay (LFI) and *α*-trapLFI sensor. Images of conventional LFI (left); *α*-trapLFI without a polyvinyl alcohol (PVA) tape (middle); and *α*-trapLFI with PVA tape (right). The strong signal is shown in an *α*-trapLFI with PVA tape compared to a conventional LFI, and no signal is shown in an *α*-trapLFI without PVA tape. All images were obtained using a ChemiDoc XPS+ imaging system (Bio-Rad).

**
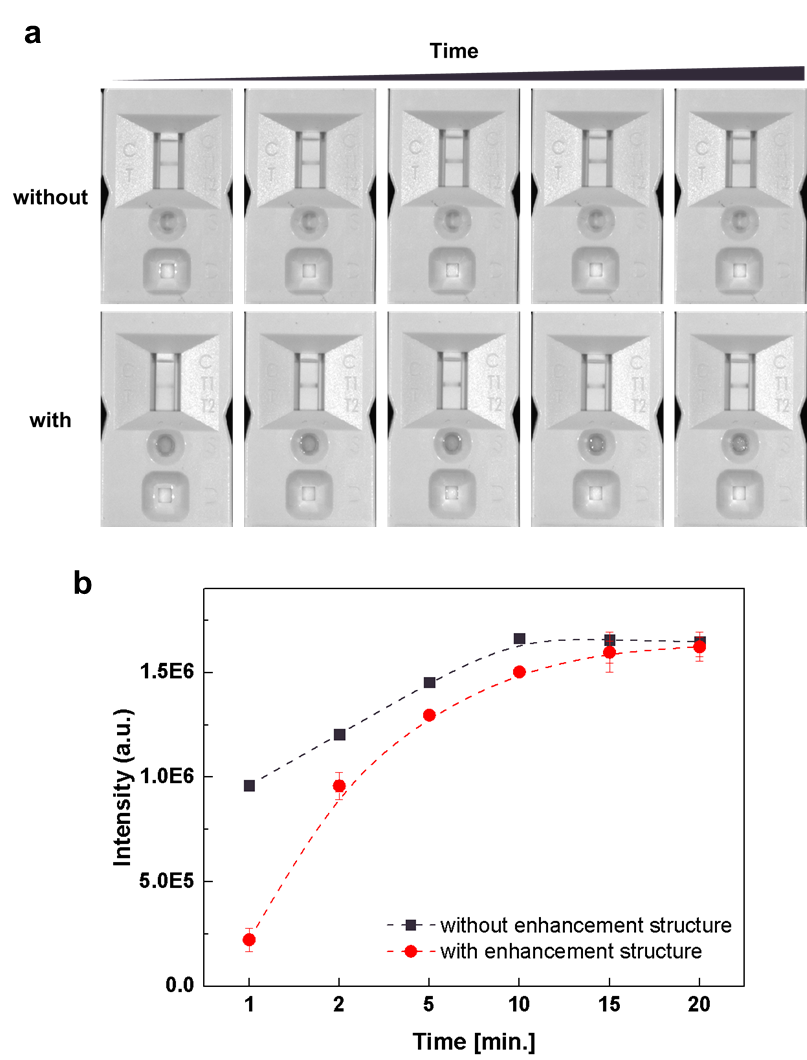
**

**Supplementary Fig. S5**. Effect of PVA tape on immunoassay. (a) Images of immunoassay without or with PVA tape; and (b) Intensity of immunoassay without or with PVA tape. All images were obtained using a ChemiDoc XPS+ imaging system (Bio-Rad), and the band intensities were measured using Image Lab software (version 6.1, <https://www.bio-rad.com/en-uk/product/image-lab-software?source_wt=imagelabsoftware_surl&ID=KRE6P5E8Z>). Error bars indicate the standard deviation from three independent experiments. Error bars indicate the standard deviation from three independent experiments.

**
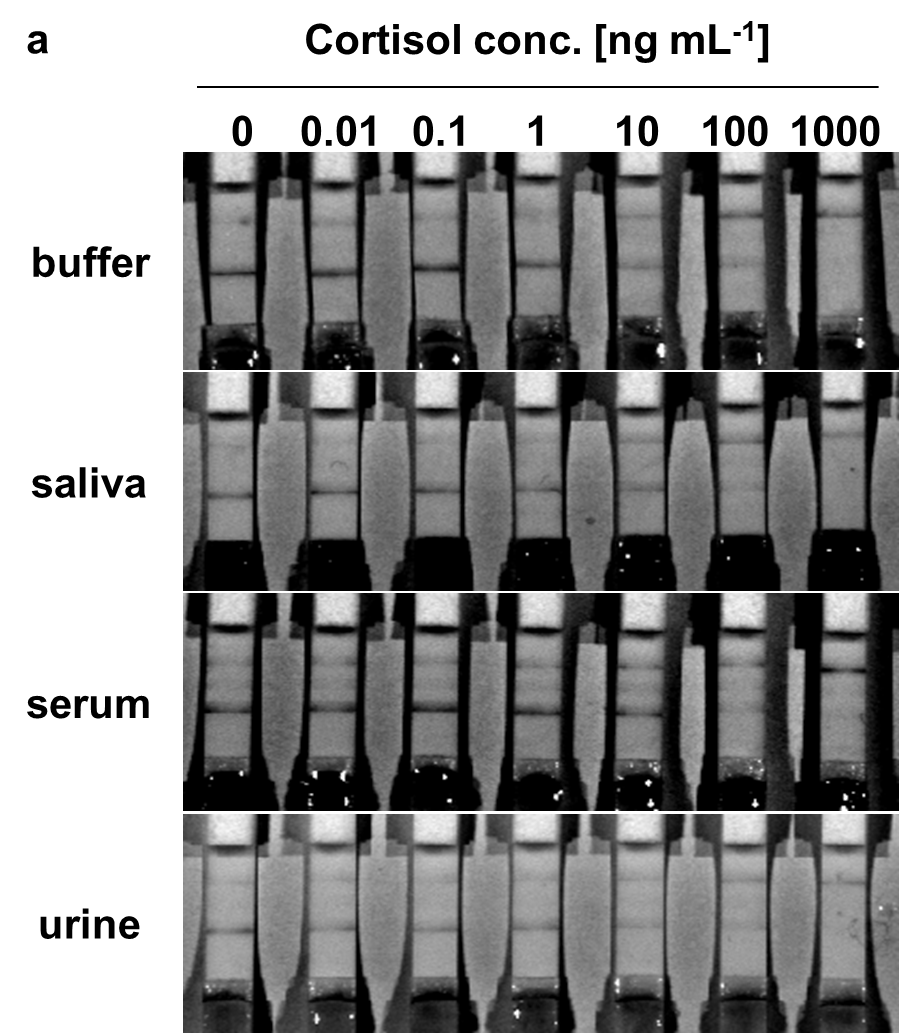

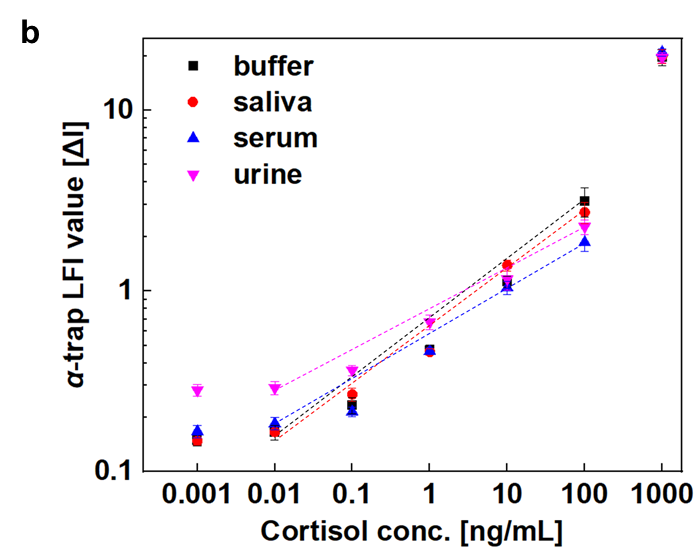
**

**Supplementary Fig. S6.** Sensitivity of *α*-trapLFI sensor for (a) analysis of cortisol in human body fluids including saliva, serum, and urine. (b) The signal intensity was linear from 0.01 to 100 ng∙mL^-1^ for all bodily fluids with an *R*^2^ of 0.9704, 0.9777, 0.9672, 0.9785 for buffer, saliva, serum and urine sample. All images were obtained using a ChemiDoc XPS+ imaging system (Bio-Rad), and the band intensities were measured using Image Lab software (version 6.1, <https://www.bio-rad.com/en-uk/product/image-lab-software?source_wt=imagelabsoftware_surl&ID=KRE6P5E8Z>). Error bars indicate the standard deviation from three independent experiments.


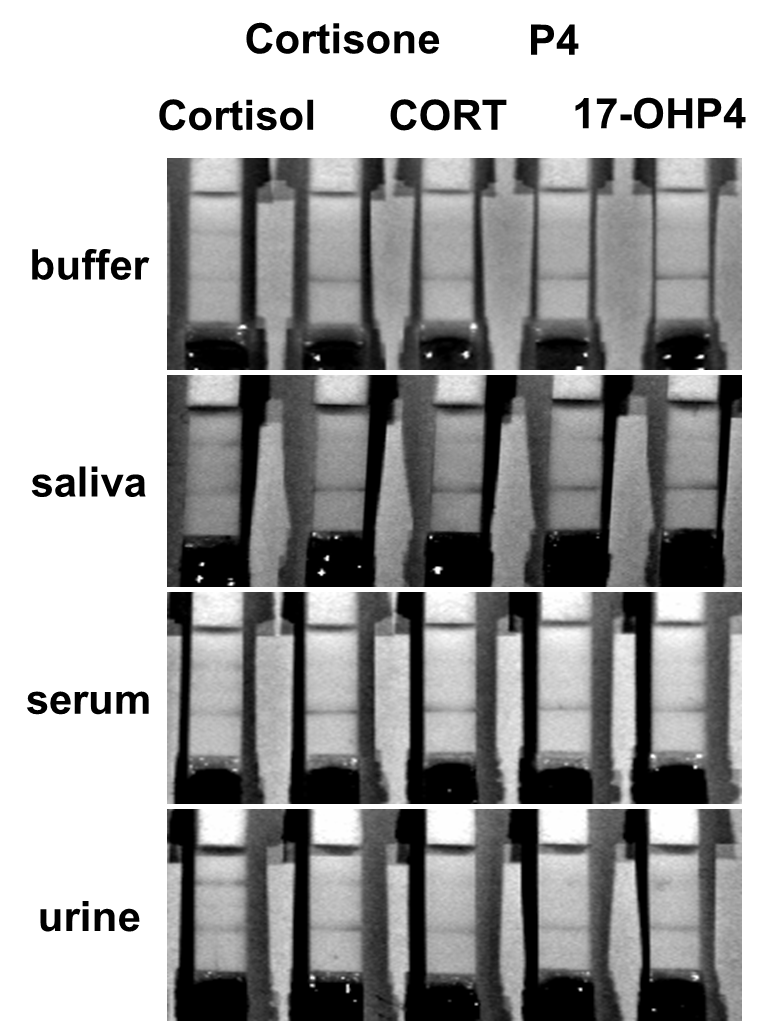


**Supplementary Fig. S7.** Cross-reactivity of *α*–trapLFI sensor in human body fluids including saliva, serum, and urine. All images were obtained using a ChemiDoc XPS+ imaging system (Bio-Rad).
